# Metacognition, Desire Thinking and Craving in Problematic Video Game Use

**DOI:** 10.1007/s41347-022-00272-4

**Published:** 2022-08-22

**Authors:** Jack Bonner, Andrew Allen, Mary Katsikitis, Steven Love, Lee Kannis-Dymand

**Affiliations:** 1 School of Health and Behavioural Sciences, University of the Sunshine Coast, 90 Sippy Downs Drive, Sippy Downs, QLD 4556 Australia; 2 Road Safety Research Collaboration, University of the Sunshine Coast, 90 Sippy Downs Drive, Sippy Downs, QLD 4556 Australia; 3 College of Education, Psychology and Social Work, Flinders University, Stuart Road, Bedford Park, SA 5042 Australia

**Keywords:** Metacognition, Desire thinking, Internet gaming disorder, Video games, Addiction

## Abstract

Gaming addiction is now a prevalent and persistent phenomenon in modern society. This study aimed to assess the metacognitive model of desire thinking and craving in explaining problematic video game use and to examine the role that specific motives for gaming have towards positive metacognitions about desire thinking. A sample of participants (*N* = 186) aged between 18 and 58 years old, who were primarily male (81.8%), played online games and met the inclusion criteria for Internet gaming disorder, completed an online survey, and the data were cross-sectionally analysed. Specifically, path analysis confirmed that the metacognitive model of desire thinking and craving was predictive of gaming-related cognitions and cravings. Furthermore, an expanded model, which included measures of psychopathology, suggested that anger and anxiety may magnify the driving metacognitive and cognitive processes underlying cravings. Finally, a multiple regression revealed that gaming for escapism, coping and skill development associated with stronger positive metacognitions about desire thinking. The findings of this study reinforced the importance of understanding motive when exploring problematic gaming and provided support for the role of metacognitions about desire thinking in shaping video game use cravings. Such findings could benefit both research and practice in their approach to understanding and intervening on problematic gaming behaviours.

## Introduction

Video game use is a prevalent activity across many cultures. The proportion of people across all ages who play video games was estimated to be 67% in Australia, with 47% of gamers being female (Brand et al., 2019), and 65% among adults in the USA (Entertainment Software Association, 2019). Between 1.1 and 10.9% of the gaming population are ‘highly engaged’ users without their use being inherently problematic (Van Rooij et al., 2018). However, researchers in the field agree that a proportion of gamers display a problematic pattern of gaming, which is worthy of study (Ferguson & Colwell, 2020). Global health organisations have demonstrated increasing awareness of this pattern of gaming, with the International Classification of Diseases (11^th^ edition) offering a diagnostic category of Gaming Disorder (World Health Organization, 2019), and the DSM-5 now containing *Internet gaming disorder* (IGD) as a mental health condition (American Psychiatric Association, 2013).

According to the DSM-5 (American Psychiatric Association, 2013), individuals must meet five of the following nine criteria in the past 12 months to be diagnosed with IGD: a preoccupation with gaming, withdrawal symptoms when gaming is not possible (e.g., anxiety, sadness), the development of a tolerance (more gaming is required to satisfy urges), difficulties in reducing gaming, a reduction in activities outside of gaming, continuing to game despite the associate problems, deceiving family or peers about the amount of time spent gaming, using gaming to relieve negative moods and a history of jeopardising jobs or relationships due to gaming. IGD has estimated prevalence rates of 1.0% among young adults (18–24 years) and 0.5% among all adults, highlighting its low prevalence in the population (Przybylski, et al., 2017). Despite the low estimated rates of diagnosis, individuals with *problematic video game use* (PVGU) have been found to have increased functional impairments, such as sleep disorders and obesity, and further psychopathology, such as increased symptoms of depression, anxiety, aggression and problematic Internet pornography use (Ko et al., 2020; Stockdale & Coyne, 2018).

While previous research has shown that the severity of PVGU is related to particular genres of gaming (e.g., massive multiplayer online role-playing games; Elliott et al., 2012; Na et al., 2017), the motivation for engaging in gaming has demonstrated to be a more robust predictor of IGD, even when compared to genre and underlying psychopathology (Laconi et al., 2017). Specifically, it has been evidenced that using gaming to escape reality, for competition purposes, for coping with stress and for social motives, is predictive of IGD symptom severity and may somewhat mediate the relationship between IGD symptomology and subsequent psychiatric symptoms (Ballabio et al., 2017; Laconi et al., 2017). Such dimensions of motivation are thought to impact on problematic addictive behaviours and thus psychopathology because they reflect a higher positive appraisal of the behaviour, regardless of whether it is being used adaptively or not. In addition to these motives, motivation for skill development and recreation have also been identified, although these are more ambiguous in their relation to IGD symptoms (Ballabio et al., 2017; Demetrovics et al., 2011; Laconi et al., 2017).

Various studies have illustrated that comparisons may be made between PVGU and established behavioural addictions. For example, PVGU has also demonstrated similarities with substance dependence and other behavioural addictions (e.g., gambling) on neuropsychological levels (Han et al., 2011; Hellman et al., 2013). Specifically, it has been shown that alike other addictive disorders, the activation of parietal brain regions linked to emotional processing was present among individuals with PVGU when they were presented with game-related cues (Thalemann et al., 2007). In addition, there has been evidence in the increased involvement of the dopaminergic system among individuals with PVGU, in that increased Internet video games use and the desire to play video games were positively associated with increased activation of reward systems (Han et al., 2011). It has also been identified gaming addiction was found to be the strongest predictor of compulsive Internet addiction (Van Rooij et al., 2010), highlighting the particularly debiliating nature of gaming addiction in particular. As such, PVGU can itself be defined as a specific and problematic behavioural addiction (Király et al., 2014).

Craving is an important feature of addictions involving substance use (Tapper, 2018) and also plays a role in behavioural addictions, such as gambling (Limbrick-Oldfield et al., 2017). To further explore PVGU in the context of a behavioural addiction, it is thus important to examine factors relating to craving of video game use. The *elaborated intrusion theory* suggests that craving is shaped by cognitive processes that occur subsequent to external cues, and these processes may elaborate on cues by selectively attending to target-related information, such as memory of engaging in the target behaviour (Kavanagh et al., 2005). These elaborative processes have been conceptualised as desire thinking, which describes an individual’s voluntary engagement in the elaboration of both verbal (e.g., persistent self-talk) and imaginal (e.g., imagining or remembering) thoughts that relate to and encourage engagement in a desired target (Caselli & Spada, 2010, 2011). The influence of desire thinking towards craving has been identified for individuals with problematic alcohol use, smoking and gambling (Caselli & Spada, 2010), as well as for subclinical behavioural addictions (Caselli & Spada, 2013). Beyond associations with craving, desire thinking is more broadly associated with the addictive behaviours of gambling and Internet use (Mansueto et al., 2019). Furthermore, desire thinking has demonstrated to be a defining factor in the processes involved in an initial urge to play a video game to the end decision to play, despite the presence of an important and conflicting non-gaming activity (Brandtner et al., 2020).

Desire thinking is explained to be problematic when it becomes perseverative and dysregulated. Metacognitions are a self-regulatory component that has shown to play a role in the dysregulation of various processes, including desire thinking (Caselli & Spada, 2013; Wells & Matthews, 1994). Metacognition refers to the cognitive processes that monitor, appraise and influence other lower-level cognitions (Flavell, 1979). From a clinical perspective, the *cognitive attentional syndrome* (CAS) refers to a pattern of extended thinking in which negative thoughts are preserved and more easily accessed (Spada et al., 2013; Wells & Matthews, 1996). According to Wells (2009), the CAS is both the product and result of unhelpful metacognitive beliefs concerned with the function and controllability of thought processes. Metacognitive beliefs, about desire thinking specifically, are therefore concerned with perceptions about the function and control about desire thinking processes and are thought to regulate experiences of craving (Caselli & Spada, 2011, 2013).

**Fig. 1:**
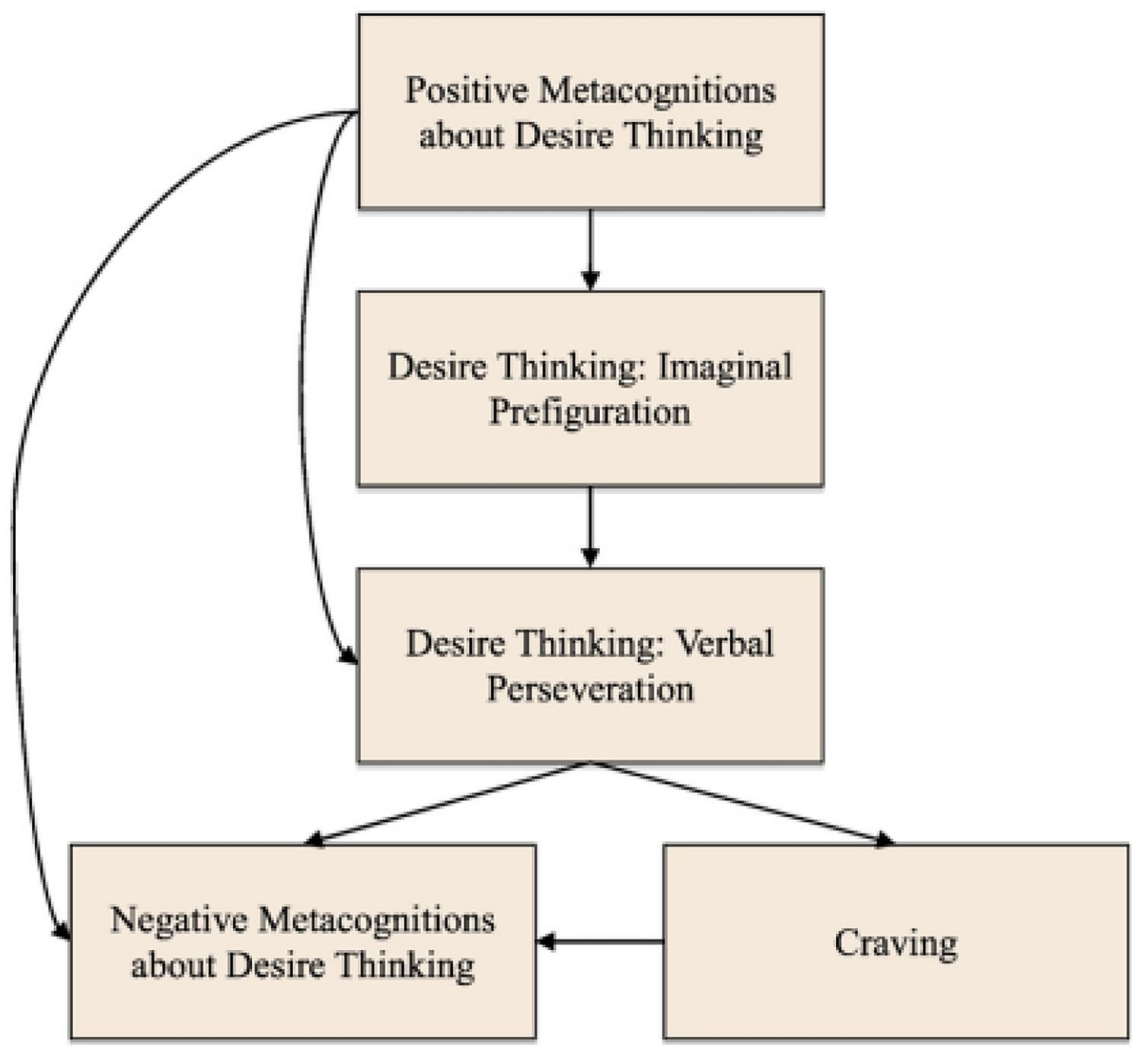
The metacognitive model of desire thinking and craving. Reprinted from Caselli and Spada (2015)

Metacognitive beliefs that impact desire thinking can be inherently positive or negative. Where positive beliefs may reflect perceived benefits of desire thinking, negative beliefs may relate to the perceived danger or uncontrollability of desire thinking (Caselli & Spada, 2013). The relationships between metacognitions and components of desire thinking have been explained in a proposed *metacognitive model of desire thinking and craving* (MMDTC; see Fig. 1). This model posits that positive metacognitions about desire thinking lead to an increased propensity to use and depend on desire thinking processes (i.e., imaginal prefiguration and verbal perseveration), and thus, stronger cravings towards the behaviour. This hierarchical relationship composing of thought processes that increases both the cognitive and physiological experiences of cravings is then thought to intensify negative metacognitive beliefs about the uncontrollability and consequences of desire thinking, further increasing CAS activity (e.g., desire thinking) and an aversive feedback loop (Caselli & Spada, 2015).

The MMDTC has demonstrated that cognitive elaboration is relevant for several addictive behaviours, through observed good model fit in samples of problematic alcohol use, gambling, Internet use and smoking (Caselli & Spada, 2015). It was also later evidenced as being a valid predictor for Internet pornography use, both in its original form and through an expanded model, in which positive metacognitions about desire thinking had a direct positive relationship with anxiety, and verbal perseveration had a direct positive relationship with depression, anger and anxiety (Allen et al., 2017, 2021). It was explained that the negative emotions may exacerbate the experience of cravings (to alleviate the negative affect) and, thus, the negative metacognitive beliefs about the danger of desire thinking. Previous studies have demonstrated that those with gaming addiction report elevated levels of anxiety, depression and anger compared to a healthy control group (Stockdale & Coyne, 2018), and thus, the expanded MMDTC may be translatable to the dynamics of gaming addiction.

### The Present Study

Given the conceptualisation of PVGU as an addictive behaviour, exploring the validity of the MMDTC may help determine whether craving in PVGU is also driven by elaborated cognitive processes. However, research to date has not examined this model in the context of PVGU, despite its growing prevalence. Furthermore, an exploration of how various psychopathologies interact with the cognitive components of this model (as per Allen et al., 2017) may lead to a more thorough understanding of how cravings are strengthened. Finally, little research has examined the relationship between motivations for gaming and the influence this has towards positive metacognitions about desire thinking. Such findings could identify those at particular risk for developing dysfunctional metacognitive beliefs that influence the frequency of desire thinking processes and thus more prone to experience gaming-related cravings.

Based on the limitations in the current literature, the present study therefore primarily aimed to explore the applicability of the MMDTC across various dimensions of PVGU. Specifically, three research aims were developed: (a) to assess the validity of the MMDTC for individuals with PVGU, (b) to examine how various psychopathologies (i.e., depression, anxiety and anger) may interact within the framework of the MMDTC, and (c) to detect whether any specific motives were associated with positive metacognitions about desire thinking. This relationship was explored because such positive metacognitive beliefs reflect positive appraisals of thoughts about gaming, which may then relate to the positive appraisal of gaming in general (as reflected though different forms of motivation). The partial overlap between positive metacognitions and some motives has been observed previously (Marino et al., 2020), and similar relationships have been identified in smoking (Nikčević et al., 2017).

## Method

### Participants

Following institutional ethical approval, an online survey was shared on various online social media groups (i.e., Facebook groups) and forums dedicated to video games (i.e., Reddit). Following a research project information sheet, which detailed components of the study and matters of confidentiality, participants were presented with screening questions, which only allowed participants to continue the survey if they were 18 years or older and had played video games in the past 12 months. In total, 2247 respondents completed the survey; however, only 186 cases in the data met the IGD inclusion criteria, in that they endorsed at least five out of the nine IGD criteria as reflected by a score of at least 5 in the Internet Gaming Disorder Test (Kiraly et al., 2017). All data were anonymous and transferred into SPSS for analyses. A large majority (81.8%) of the IGD sample was male, and the ages ranged from 18 to 58 years (*M* = 24.11, SD = 7.88). Approximately a quarter (26.3%) of the sample was from the USA, 11.7% from the UK, 4.7% from Australia, 4.7% from Canada, 4.1% from India and 3.5% from Brazil, and a further 45.0% was made of participants from various other countries. More than half of the sample (60.3%) was Caucasian, 13.8% Asian, 10.1% Hispanic, 6.3% Indian, 2.5% Arab, 1.9% of African descent and a remaining 5.1% from other ethnicities.

In regard to gaming, participants reported that the number of hours per week spent on gaming ranged from 4 to 105 h (*M* = 38.67, SD = 21.86). The most played genre in the sample was multiplayer online battle arena (46.8%), followed by first person shooter (17.5%), massive multiplayer online role-playing game (9.9%), survival (7.0%), strategy (5.3%), role-playing game (4.7%), action (4.7%) and other (4.1%).

### Measures

#### Internet Gaming Disorder

The *Internet Gaming Disorder Test* (IGDT-10; Kiraly et al., 2017) was used to screen for symptomology associated with IGD. The IGDT-10 contains 10 questions (e.g., “have you played to relieve a negative mood (for instance helplessness, guilt, or anxiety?”), which are scored on a 3-point scale relating to how often the participant engaged in the behaviour, where 1 = never and 3 = often. To obtain a score that reflected IGD symptom criteria, the scores were summed, where only scores of 3 were counted as 1 point. Five points out of a possible ten were indicative of PVGU. The IGDT-10 has suitable reliability and validity (*α* = .81) and adequate invariance across language and gender (Király et al., 2019).

#### Video Game Craving

Craving was measured using a modified version of the *Penn Alcohol Craving Scale* (PACS; Flannery et al., 1999). Specifically, the item structure of the PACS was maintained, although the focus of the items was directed towards video game use in the past week, rather than alcohol cravings. The scale contained five self-report items that measured factors of video game craving during the previous week (e.g., “at its most severe point, how strong was your craving during this period?”). Each item is scored on a dynamic 7-point scale, with higher scores reflecting increased craving. The PACS was modified in line with previous studies that used similar modifications (Caselli & Spada, 2013). The original scale has demonstrated excellent internal reliability (*α* = .92) and good construct, discriminant and predictive validity (Flannery et al., 1999).

#### Desire Thinking

The *Desire Thinking Questionnaire* (DTQ; Caselli & Spada, 2011) was used to measure desire thinking across ten items and two subscales. The first subscale, verbal perseveration, measures how verbal thoughts about desire-related content and experience persevere (e.g., “I mentally repeat to myself that I need to practice the desired activity”). The second subscale, imaginal prefiguration, measures the extent to which mental images about desired content are prefigured (e.g., “I imagine myself doing the desired activity”). Items were adapted to specify thoughts about video game use, with ratings on a 4-point scale ranging from 1 (*almost never*) to 4 (*almost always*). Score ranges for both subscales are between 5 and 20, with higher scores indicating increased desire thinking. Both subscales have demonstrated adequate internal consistency (*α* = .78–.80) as does the total score (*α* = .83; Caselli & Spada, 2011).

#### Gaming Motives

The 27-item *Motives for Online Gaming Questionnaire* (MOGQ; Demetrovics et al., 2011) provided a measure of motivation to play video games across seven motivational dimensions: escape (escaping from reality), coping (with stress), fantasy (fulfilling specific desires), skill development (e.g., attention and coordination), recreation (enjoyment), competition (competing with others) and social (building relationships with others). Participants are asked to rate each self-report item on how frequently different motives were a factor in their gameplay (e.g., social; “I play online games… because I can get to know new people”). Items were rated on a 5-point scale assessing frequency (1 = “never/almost never”; 5 = “almost always/always”). Higher scores reflect increased motives. This scale has demonstrated a satisfactory internal consistency (*α* = .79–0.90; Demetrovics et al., 2011; Kim et al., 2016).

#### Metacognition

Metacognitive beliefs about desire thinking were measured using a modified version of the *Metacognitions about Desire Thinking Questionnaire* (MDTQ; Caselli & Spada, 2013), in which the items were adapted to specify desire thoughts about video game use rather than a “desired activity/object”. The MDTQ portrays three types of metacognitions across 18 items: (a) positive metacognitions about desire thinking (e.g., “I need to think about playing a video game in order to feel motivated”), (b) negative metacognitions about desire thinking (e.g., “I cannot stop thinking about playing a video game once I get started”) and (c) need to control desire-related thoughts. For the current study, the third subscale was not used in line with prior research (e.g., Caselli & Spada, 2015), as the concept was outside of the scope of this study. A 4-point scale of agreeableness (1 = “do not agree”; 4 = “agree very much”) is used. Higher scores reflect increased endorsement of metacognitive belief statements. The original MDTQ has shown to possess acceptable internal consistency across its subscales (Ω = .76; Caselli & Spada, 2013).

#### Depression

Depressive symptoms were measured using the *Patient Health Questionnaire-9* (PHQ-9; Kroenke et al., 2001), which reflects depressive disorder criteria in the *Diagnostic and Statistical Manual of Mental Disorders, Fourth Edition* (DSM-IV; American Psychiatric Association, 1994). Participants are asked to rate how frequently they have experienced depressive symptoms (e.g., “feeling down, depressed, or hopeless”) in the previous 2 weeks, using a 4-point frequency scale (1 = “not at all”; 5 = “nearly every day”). The PHQ-9 has demonstrated good internal consistency (*α* = .86 to .89; Kroenke et al., 2001), test–retest reliability (*α* = .81–.96; Löwe et al., 2004) and good construct validity (Kroenke et al., 2001).

#### Anxiety

Anxiety-based symptoms were measured using the *Generalised Anxiety Disorder 7* (GAD-7; Spitzer et al., 2006), which assesses the incidence of generalised anxiety disorder symptomology with seven self-report items (e.g., “feeling nervous, anxious or on edge”). The scale is scored by rating the frequency with which participants have been bothered by GAD criteria in the DSM-IV (APA, 1994) in the previous 2 weeks. Each item is rated on a 4-point scale (1 = “not at all”; 4 = “nearly every day”), with higher scores reflecting increased symptoms of GAD. The GAD-7 has displayed good psychometric properties and internal consistency (*α* = .92; Löwe et al., 2008; Spitzer et al., 2006).

#### Anger

The incidence of anger was measured using the *Dimensions of Anger Reactions-Revised* (DAR-R; Novaco, 1975), which contains seven self-report items relating to anger experiences (e.g., “when I do get angry, I get really mad”). Participants rate the extent to which anger statements are representative of their own behaviour on a 5-point Likert scale (1 = “not at all”; 5 = “very much”). Higher scores suggest increased anger symptomology. The DAR-R has good internal consistency, test–retest reliability and validity (Kannis‐Dymand et al., 2019).

### Data Analysis

Following completion of the data collection, the data were imported into SPSS (version 27) for inspection of the adequacy of the data for analyses. Preliminary bivariate correlations were also run to assess the strength of the individual relationships between the variables, not accounted for in the subsequent models being tested. Effect sizes of the correlations were interpreted as per guidelines by Cohen (1988), where .1 = small, .3 = medium and .5 = large. Next, to test the first and second research aims and proposed models, path analyses were implemented using SPSS AMOS (version 27). Five indices of fit were used to determine the adequacy of fit for the hypothesised models, including the chi-square (*χ*^2^) statistic, the *root mean square error of approximation* (RMSEA), the *goodness of fit index* (GFI), the *comparative fit index* (CFI) and the *standardised root mean square residual* (SRMR), as recommended by Hu and Bentler (1999). Accordingly, a significant *p* value associated with the *χ*^2^ statistic indicates model misspecification. The remaining fit indices convey ‘acceptable’ model fit, where GFI and CFI are ≥ .90 and SRMR and RMSEA are ≤ .10, or convey a ‘good’ fit using more conservative criteria, where GFI and CFI are ≥ .95, SRMR is ≤ .08 and RMSEA is ≤ .06 (Hu & Bentler, 1999). Finally, in relation to research aim 3, a multiple regression was run to test the predictive value that various gaming motives had towards positive metacognitive beliefs about desire thinking. Interpretation of effect sizes was again guided by Cohen (1988) and included Cohen’s *f*^2^ (.10 = small, .25 = medium, .40 = large) and partial *r* squared (.02 = small, .13 = medium, large = .26).

## Results

### Preliminary Analyses

To test the assumption of univariate normality, skewness and kurtosis were assessed for each variable, and the data demonstrated to be sound in all areas. Reliability analyses were also performed and indicated that internal consistency was also adequate among all the variables (*α* = .74 to .89), except for the coping and recreation motivation subscales, which were questionable (*α* = .66 to .68) and thus interpreted with caution. Notably, mean anxiety (*M* = 10.78) and depression (*M* = 14.54) scores were higher than in a prior generalised sample of Australians (*M* = 6.16 and 6.93; Kannis-Dymand et al., 2019), although lower in anger scores (*M* = 9.73 and 12.46, comparatively). As a preliminary examination of the relationships to be involved in the models, bivariate correlations indicated that there were small to large positive relationships between metacognitive beliefs with desire thinking variables (*r* = .25 to .60, *p* < .001), but only small positive correlations with the psychopathology variables (*r* = .16 to .28, *p* < .001 to .028). In addition, there were medium to large positive relationships present between depression, anxiety and anger (*r* = .45 to .69, *p* < .001). Notably, of the desire thinking variables, only verbal perseveration had a small positive relationship to anger (*r* = .19, *p* = .008). Next, video game cravings were shown to have small to large relationships with all other variables proposed to be in the MMDTC (*r* = .15 to .56, *p* < .001 to .043). Correlations involving motivational styles were sporadic, ranged from small to large (*r* = .16 to .57, *p* < .001 to .034) but were predominantly positive. Finally, for exploratory purpose, age and gender were included in the analyses and the results showed that age was negatively related to positive (*r* =  − .19, *p* = .011) and negative (*r* =  − .16, *p* = .035) metacognitions, and motivations for escapism (*r* =  − .20, *p* = .007), social reasons (*r* =  − .15, *p* = .049) and coping (*r* =  − .17, *p* = .019). Conversely, gender (female) was negatively related to gaming for competitive reasons (*r* = .32, *p* < .001). The means, standard deviations, reliability coefficients and bivariate correlations are displayed in Table 1.

**Table 1:** Descriptive statistics, reliability coefficients and correlations between the variables

Variable	M (SD)	*α*	1	2	3	4	5	6	7	8	9	10	11	12	13	14
1. PMDT	15.62 (5.10)	79	–													
2. NMDT	21.13 (6.23)	.85	.25^***^	–												
3. DT-IP	12.59 (3.36)	.74	.45^***^	.32^***^	–											
4. DT-VP	12.56 (3.48)	.76	.44^***^	.47^***^	.60^***^	–										
5. VGC	23.68 (6.53)	85	.28^***^	.41^***^	43^***^	.56^***^	–									
6. Depression	14.54 (6.58)	.83	.22^**^	16^*^	.02	.07	.16^*^	–								
7. Anxiety	10.78 (5.86)	.87	.28^***^	.25^***^	.09	.14	.22^**^	.69^***^	–							
8. Anger	9.73 (7.12)	.85	.23^**^	.16^*^	.14	.19^**^	.15^*^	.45^***^	.49^***^	–						
9. Escape	16.18 (4.26)	.89	.39^***^	.22^**^	.19^**^	.19^**^	.26^***^	.42^***^	.39^***^	.30^***^	–					
10. Social	9.67 (4.31)	.80	.26^***^	.01	.16^*^	.08	.04	.02	.04	.11	.14	–				
11. Coping	13.87 (3.73)	.66	.44^***^	.09	.32^***^	.26^***^	.19^**^	.04	.10	.09	.44^***^	.23^***^	–			
12. Competition	13.27 (4.90)	.86	.19^*^	.14	.10	13	.04	.08	.13	.22^**^	.00	.32^***^	.10	–		
13. Skills	11.62 (5.12)	.89	.40^***^	− .07	23^**^	.17^*^	.03	.03	.12	.11	.06	.48^***^	.33^***^	.47^***^	–	
14. Fantasy	12.81 (5.20)	.87	.30^***^	.12	.21^**^	.16^*^	.11	.24^**^	.28^***^	.29^***^	57^***^	.20^**^	.44^***^	− .09	.13	–
15. Recreation	13.31 (2.33)	.68	.19^**^	− .09	.22^***^	.20^**^	.18^*^	− .16^*^	− .11	.09	.11	.19^**^	.41^***^	.00	.16^*^	.25^***^
16. Age	24.11 (7.88)	–	− .19^*^	− .16^*^	− 07	− .08	− .10	− .11	− .03	− .09	− .20^**^	− .15^*^	− .17^*^	− .08	− .06	− .02
17. Gender	–	–	.02	− .09	.07	.04	.06	− .05	− .05	− .10	.03	− .05	− .04	− .32^***^	12	.02

*PMDT* positive metacognitions about desire thinking, *NMDT* negative metacognitions about desire thinking, *DT-IP* desire thinking imaginal prefiguration, *DT-VP* desire thinking verbal perseveration, *VGC* video game craving

*p < .05; **p < .01; ***p < .001

### Research Aim 1: the MMDTC and Problematic Gaming Cognitions

**Fig. 2:**
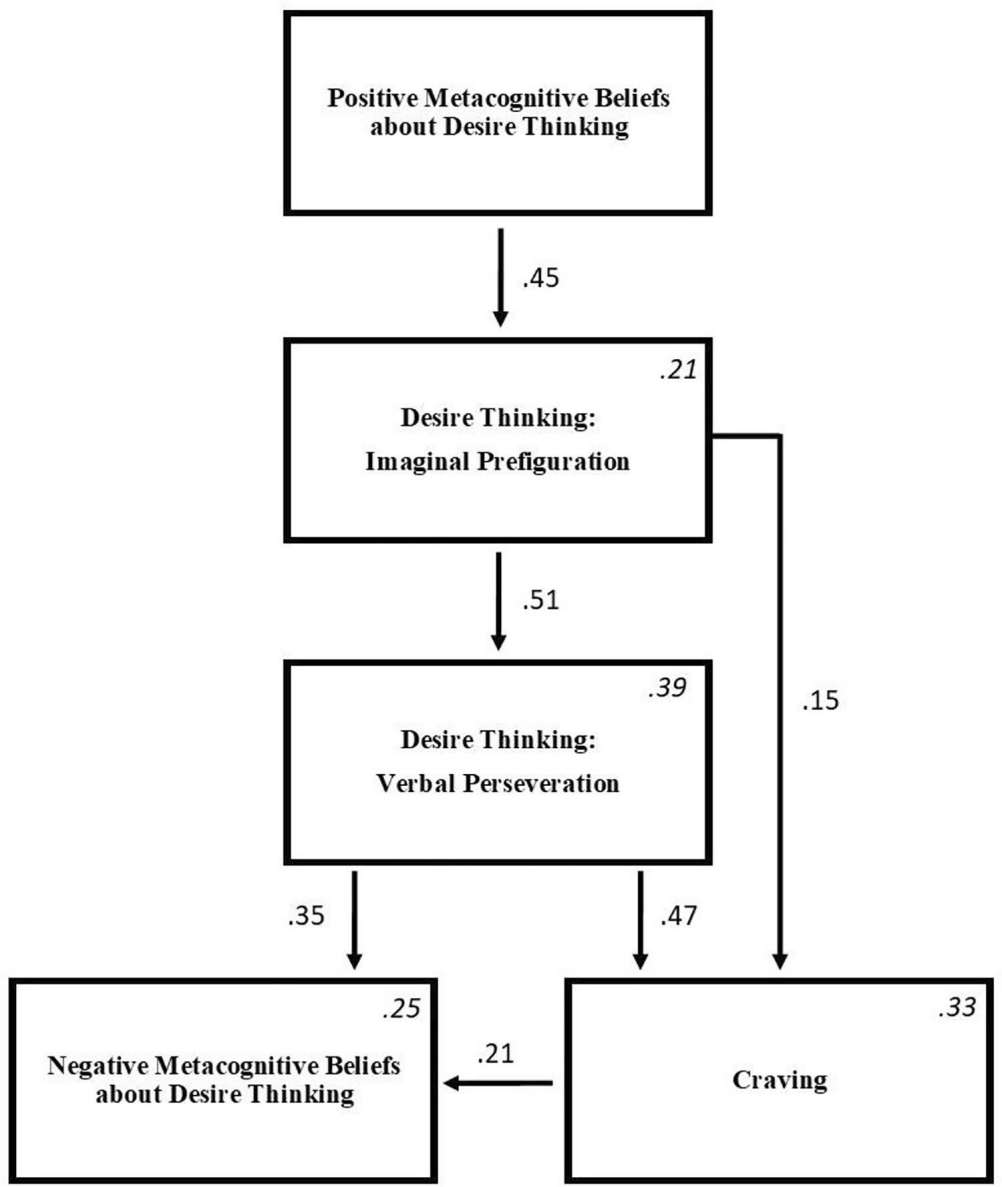
The revised metacognitive model of desire thinking and craving for PVGU

A model was drawn in SPSS AMOS as per the metacognitive model of desire thinking and craving. Given the low correlations present between age, gender and the models’ variables and the implications of producing inadequate power with the additional parameters in the model (Kline, 2005), it was decided to not control for age and gender in the path analyses. The initial results showed that the original model was a good fit to the data (*χ*^2^ (2, 15) = .21, *p* = .903, CFI = 1.00, GFI = 1.00, RMSEA = .000, SRMR = .006). Outside of the *χ*^2^ and RMSEA statistics, the standardised parameter estimates supported that the model fits the data well, except for the path between positive metacognitions and negative metacognitions (*β* = .02, *p* = .758). As per metacognitive theory (Wells & Matthews, 1996), positive metacognitions do not necessarily lead to negative metacognitions unless thinking becomes rigid and influenced by the experience of negative affect. Therefore, this constraint was removed, and the model was re-run. The revised model (Fig. 2) also displayed sound fit statistics (*χ*^2^ (3, 15) = .30, *p* = .960, CFI = 1.00, GFI = .999, RMSEA = .000, SRMR = .007), indicating that the model adequately explained dimensions of PVGU. Standardised regression weights of the revised model were all significant (*p* < .050) and ranged from small to moderate (*β* = .17 to .48), and 21 to 39% of the variance was explained among the variables.

### Research Aim 2: the MMDTC, Problematic Gaming and Psychopathology

**Fig. 3:**
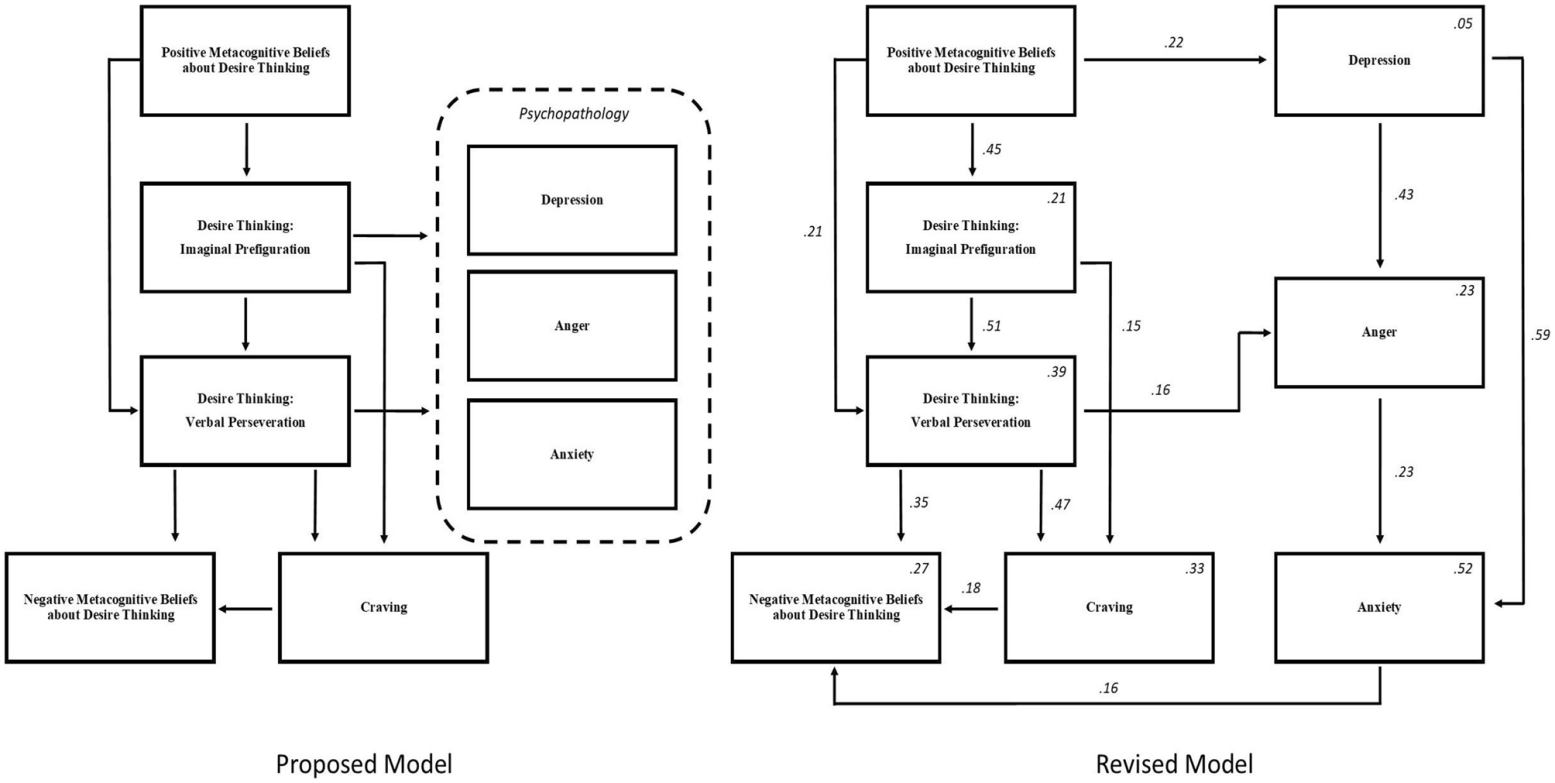
The proposed and revised metacognitive model of desire thinking, craving and psychopathology for PVGU

For the extended model including psychopathology, the initial model (Fig. 3, proposed model) was constructed to include the dimensions of anxiety, depression and anger, with all variables relating to imaginal prefiguration and verbal perseveration, as per Allen et al. (2017, 2021) initial expanded MMDTC. Initial analyses showed that inadequate fit was observed (*χ*^2^ (15, 36) = 200.53, *p* < .001, CFI = .5848, GFI = .776, SRMR = .172, RMSEA = .259). An inspection of modification indices indicated that there were strong suggestions for parameters to be added between the psychopathology variables (MI = 38.31–78–95). Given the established comorbidities between these variables, parameters were added from depression to anger and anxiety and from anger to anxiety, based on the directional relationships proposed in the previous literature (Mook et al., 1990; Painuly et al., 2005; Deschenes et al., 2012). The revised model showed an improved fit to the data (*χ*^2^ (12, 36) = 26.37, *p* = .009, CFI = .968, GFI = .967, SRMR = .074, RMSEA = .080).

Further inspections of modification indices indicated that two additional parameters were plausible. Firstly, parameters were suggested from positive metacognitions to depression. Because psychopathology was related to positive metacognitions in prior research on the MMDTC applied to pornography addiction, this parameter was added. The second suggestion was for a parameter to be added between anxiety and negative metacognitions. Given that theoretically, negative metacognitions are said to result in anxiety (Wells & Mathews, 1996), and that the presence of anxiety would theoretically increase the perceived need to control thoughts that produce that anxiety, this parameter was also added to the model. The revised model demonstrated a good fit (*χ*^2^ (10, 36) = 10.57, *p* = .392, CFI = .999, GFI = .986, SRMR = .036, RMSEA = .018).

Finally, estimates were examined to assess the proposed relationships of the model. Firstly, it was shown that the estimates indicated that as found in prior research (Allen et al., 2017, 2021), the relationship between imaginal prefiguration and the psychopathology variables was not significant. Based on these similarities with previous research, these relationships were removed. Finally, the relationships between verbal perseveration with anxiety and depression were not significant. Considering comparisons to previous research are sparse, the lack of relationships found in the current sample may implicate the unique dynamics of desire thinking in an IGD sample. Therefore, given the novel and exploratory nature of this study, these relationships were removed. The final model was shown to remain a good fit to the data (*χ*^2^ (10, 36) = 14.15, *p* = .514, CFI = 1.000, GFI = .982, SRMR = .042, RMSEA = .000). Examination of direct effects (Fig. 3, revised model) showed that small to large (*β* = .16 to .59) significant (*p* < .001 to .048) relationships were present in the model.

Following recommended bootstrap procedures (Shrout & Bolger, 2002), 1000 bootstrap samples were used to estimate the significance of indirect and total effects. The results showed that firstly, significant indirect effects were present in the model. Of note, positive metacognitions about desire thinking were shown to moderately and indirectly predict verbal perseveration, negative metacognitions, and imaginal prefiguration and video game cravings (*β* = .20 to .26), and imaginal prefiguration indirectly predicted negative metacognitions and video game cravings (*β* = .26 and .24, respectively). The indirect and total effects are displayed in Table 2. All effects were considered significant (*p* < .05).

**Table 2:** Indirect and total effects within the non-recursive model (Fig. 3)

Dependant variables	Predictor variables (*β*)						
	**PMDT**	**DT-IP**	**DT-VP**	**Depression**	**Anger**	**Anxiety**	**VGC**
**Standardised indirect effects**							
DT-VP	.23^**^						
Anger	17^***^	.02^*^					
Anxiety	.17^***^	.08^**^	.04^*^	.10^***^			
VGC	.27^***^	.24^***^					
NMDT	.23^***^	.25^**^	.09^*^	.11^*^	.04^*^		
**Standardised total effects**							
DT-IP	.45^**^						
DT-VP	.44^**^	.51^**^					
Depression	.22^**^						
Anger	.17^***^	.08^*^	.16^*^	.44^**^			
Anxiety	.17^***^	.02^**^	.04^*^	.69^**^	.23^***^		
VGC	.27^***^	.39^**^	.47^***^				
NMDT	.23^***^	.11^**^	.44^**^	.11^*^	.04^*^	.16^*^	.18^*^

*PMDT* positive metacognitions about desire thinking, *NMDT *negative metacognitions about desire thinking, *DT-IP* desire thinking imaginal prefiguration, *DT-VP* desire thinking verbal perseveration, *VGC* video game craving

*p < .05; **p < .01; ***p < .001

### Research Aim 3: Motivations for Gaming and Positive Metacognitions

A hierarchical multiple regression was used to evaluate whether the motives of escape, fantasy, coping, skill development, social, recreation and competition predicted the strength of positive metacognitions about desire thinking, while controlling for gender and age. Using the enter method, gender and age were entered in step 1 and the model significantly predicted 3.9% of the variance in positive metacognitions (*F* (2, 181) = 3.61, *p* = .029, *R* = .197). In step 2 which included the motivation variables, the total model explained 33.7% of the variance in positive metacognitions (*F* (9, 181) = 9.70, *p* < .001, *R* = .580). At the univariate level, several motivational forms were found to be significant predictors of positive metacognitions about desire thinking, including escape (*β* = 28, *t* = 3.36, *p* < .001, *r*^2^ = .06), coping (*β* = .19, *t* = 2.27, *p* = .024, *r*^2^ = .03) and skill development (*β* = .29, *t* = 3.54, *p* < .01, *r*^2^ = .07). The total effect of the model was considered large (*F*^2^ = .51), while the individual effects were small to medium in size, explaining 2.9 to 6.8% of the variance in positive metacognitions. The statistics for the regression are included in Table 3.

**Table 3:** A multiple regression analysis with gender, age and gaming motives predicting positive beliefs about desire thinking

Motivational variables	*Effects towards PMDT*					
	***B***	**SE**	***β***	***t***	***p***	***r***^***2***^
1. Gender	.30	1.04	.02	.29	.771	.00
2. Age	− .13	.05	− .20	− 2.68	.008	.04
1. Gender	.56	.94	.04	.59	.555	.02
2. Age	− .06	.04	− .09	− 1.33	.184	.01
3. Gaming to escape reality	.33	.10	.28	3.36	< .001	.06
4. Gaming for social reasons	.01	.09	.01	.15	.881	.00
5. Gaming to cope with stress	.26	.11	.19	2.27	.024	.03
6. Gaming for competition	.02	.08	.02	.30	.767	.00
7. Gaming for skill development	.29	.08	.29	3.54	< .001	.07
8. Gaming to fulfil fantasies	.00	.08	.00	.05	.963	.00
9. Gaming for recreation	.04	.16	.02	.25	.799	.00

## Discussion

The present study examined the validity of the MMDTC and the expanded metacognitive model of desire thinking, craving and psychopathology for PVGU. Path analyses were used to test the adequacy of the models among a sample of online gamers presenting IGD criteria. Furthermore, it was aimed to investigate whether different forms of motivation for gaming predicted positive metacognitions about desire thinking (and thus the MMDTC for gaming).

### The Metacognitive Model of Desire Thinking and Craving for Gaming

With regard to the first research aim, the findings were in support of the MMDTC explaining variance in cravings specific to a gaming context. As such, analyses indicated that positive metacognitions about desire thinking had a direct impact on imaginal prefiguration and verbal perseveration, reinforcing the role that metacognitive interpretations play in the selection or engagement of the subsequent thought processes. Imaginal prefiguration was also shown to have a direct impact on verbal perseveration, supporting the notion that gaming imagery leads to gaming-related self-talk (Caselli & Spada, 2010, 2011). The direct impacts of verbal perseveration on craving and negative metacognitions about desire thinking provide evidence for the presence of the CAS for PVGU (Caselli & Spada, 2015; Wells & Matthews, 1994, 1996), in that once a relationship between the development of cravings and the desired behaviour (i.e., gaming) has been established, the associated negative metacognitive beliefs surrounding desire thinking and the behaviour are re-enforced and embedded in the self-regulatory process.

Next the findings indicated that there was no significant direct pathway between positive and negative metacognitions about desire thinking. While prior studies on metacognitions about desire thinking have shown relationships between positive and negative metacognitions in a variety of models predicting addictive behaviours (i.e., alcohol use, Internet use, gambling disorder, tobacco use; Caselli & Spada, 2015), the current study implements a gaming-specific version of the scales that were administered to gamers meeting certain criteria for IGD, which may have impacted on the results. Nonetheless, there was a significant indirect effect of positive metacognitions towards negative metacognitions, through effects of desire thinking and cravings, which is more representative of theoretical processes presented in the *self-regulatory executive function* (S-REF; Wells & Mathews, 1994, 1996) model. Specifically, positive metacognitive beliefs about thought processes representative of the CAS are thought to increase the frequency of dysfunctional thoughts, reducing executive control and thus contributing to the perception of low cognitive control. This finding is consistent with prior metacognitive research on other behaviours outside of problematic gaming behaviours (Allen et al., 2017; Caselli & Spada, 2013, 2015; Wells, 2009).

The findings also revealed a direct relationship between imaginal prefiguration and craving, which highlighted that both factors of desire thinking impacted craving, consistent with previous findings that both imaginal prefiguration and verbal perseveration predicted craving levels for alcohol use, Internet use, gambling disorder and tobacco use (Caselli & Spada, 2011, 2015). This is also supported by findings demonstrating that a manipulation of imagery impacts craving and its associated affect (Bywaters et al., 2004). One further possibility is that the level of interactivity and immersion that may be experienced while gaming, or thinking about gaming, may result in especially vivid imagery that translates more easily into craving experiences. Finally, video game craving had a direct effect on negative metacognitions about desire thinking, suggesting that an increased craving experience is evaluated as a lack of control over desire thoughts and gaming, consistent with other clinical samples (Caselli & Spada, 2015).

### The Extended Metacognitive Model of Desire Thinking and Craving for Gaming

When the model incorporated depression, anxiety and anger, the proposed expanded MMDTC for PVGU was only partially supported, in that, firstly, imaginal prefiguration was not related to psychopathology (as per previous research; Allen et al., 2017, 2021), and secondly, only verbal perseveration had a significant direct relationship with anger. This finding aligned with some previous literature, which explains that metacognitions guide the selection of perseverative cognitive strategies like anger rumination (Salguero et al., 2020). The relationships identified between desire thinking and anger may then reflect the distinct levels of engagement with target related information across desire thinking factors. That is, imaginal prefiguration may be a more transient cognitive occurrence with more impactful indirect influences towards craving, while verbal perseveration may be longer lasting and more observable in behaviour. It may also lead to anger where there is a conflict between the presence of this self-talk and other important activities that conflict with gaming (Brandtner et al., 2020).

The lack of relationships between verbal perseveration with anxiety and depression also potentially highlight the unique interactions between the MMDTC with psychopathology in a IGD related sample, warranting further investigation to better understand the dynamics that these factors play within gaming-related cravings. This is alike the relationships between anxiety, anger, and depression, in that past research has highlighted the complex interactions between these psychological presentations (Mook et al., 1990; Painuly et al., 2005; Deschenes et al., 2012), but this dynamic is again unknown in the domain of gaming-related cognition. In the case of the present findings, anger acts as a mediating factor between desire thinking and experiences of anxiety, and thus the perceptions about the danger and uncontrollability of desire thinking.

As illustrated, positive metacognitive beliefs were found to predict depression, while anxiety was found to be predictive of negative metacognitive beliefs. As per the S-REF theory (Wells & Matthews, 1994, 1996), positive metacognitive beliefs are only inherently dysfunctional, when a CAS, which involves a pattern of focused and extended thinking (e.g., desire thinking), contributes to the perseveration of negative thoughts and affect (e.g., psychopathology) and the resulting negative metacognitive beliefs (Wells, 2009). Specifically, anxiety has prominently linked to the occurrence of negative metacognitive beliefs (Wells, 2009). The relationship between positive metacognitive beliefs and depression is also one that has been highlighted in alternate research on beliefs about depressive rumination (Salguero et al., 2021) and may be explained by an understanding that one is dependent on gaming-related desire thinking, which may lead to a feeling of helplessness and thus depressive-related thoughts.

The path analysis representing the expanded metacognitive model of desire thinking, craving and psychopathology for PVGU contained indirect effects that were all positive and significant. This reflected that the elaborative cognitive-affective processes involved do not necessarily occur in a linear fashion but are more comprehensive in nature. A final consideration is that the effects of the relationships varied from small to large, thus suggesting that different variables may have varying impacts within the MMDTC. In particular, the relationships between psychopathology and the original MMDTC variables were relatively weak. This may have been because a specific IGD sample was used and thus the variability of responses was limited, although further research is needed to confirm the roles that these factors play in the process of gaming addiction.

### Motivations for Gaming and Metacognitions About Desire Thinking

The final research aim examining motivational and positive metacognitions highlighted several motivations for gaming that may increase the strength of positive metacognitions about desire thinking, and thus the activation of desire thinking. Specifically, the motives of escape, coping and skill development accounted for a small amount of the variance in positive metacognitions about desire thinking. This may indicate that positive appraisal of gaming in terms of its benefits of providing a sense of escape, and of facilitating coping with negative affect, has an impact on positive appraisal of desire thoughts about gaming. A discrepancy between the bivariate correlations and the multivariate analysis also indicated that while stronger motivation in any regard may increase positive metacognitions about desire thinking, only escape, coping and skill development motivational dimensions present unique variance in predicting positive appraisals of desire thinking about gaming.

While such results preclude previous findings, the current bivariate analyses and prior studies (Ballabio et al., 2017) indicate that using gaming as a means of escapism or coping is significantly related to problematic online gaming indicating the motivational types are dysfunctional in nature. Although further research is required, such findings also suggest that different motivational types may also potentially determine whether the associated positive metacognitions are inherently adaptive or not. As discussed by Love et al., (2019, 2021), positive metacognitions may be used adaptively in the context of skill development and performance to momentarily increase self-regulatory resources towards a particular stimulus, or maladaptive when processes are influenced by negative affect and ineffective coping strategies.

### Implications

The present study provided initial support for the utility of a metacognitive model of desire thinking, craving, anger and anxiety for PVGU, while reinforcing the metacognitive framework as a way of understanding addictive behaviours. Results are mostly aligned with research of similar models among samples of other addictive behaviours, which may have both theoretical and clinical implications for the conceptualisation of PVGU as an addictive behaviour. The results also provide support that metacognitions about desire thinking and desire thinking may be central cognitive processes in craving, anger and anxiety for individuals with PVGU. This therein highlights the utility of future research into therapeutic interventions targeting and modifying dysfunctional metacognitive beliefs that increase the frequency (positive beliefs) and intensity (negative beliefs) about desire thinking in the context of PVGU. Where desire thinking may be problematic for cravings and associated psychopathology, it is metacognitions that precede and maintain these thinking styles. These approaches are consistent with metacognitive therapy for other addictive behaviours (Caselli et al., 2018; Palmieri et al., 2020; Spada et al., 2013; Wells, 2009), such as therapy that aims to reduce the negative impact of these processes (e.g., attention training; Wells, 2009).

Finally, the findings of this study highlighted that specific types of motivation are associated with not only gaming-related desire thinking and cravings but are predictive of the metacognitive beliefs that influence these processes. Future research may benefit from further investigation of these relationships and possibly look to expand the current MMDTC to incorporate how motivation may play a role in the development and maintenance of desire thinking. Furthermore, motivational types may give practitioners a context for the development of metacognitive beliefs and allow for a more tailored intervention approach.

### Limitations

Several limitations must be considered when interpreting these findings. A significant limitation is that the analyses (i.e., path analysis; regression) were correlational by nature and involved variables that were measured through self-report methodologies, which meant that causation could not be inferred. Furthermore, alternative pathway structures between the variables may be explained by different theoretical frameworks. Another limitation involved the use of convenience sampling for recruitment, and that the subsequent over-representation of males (84.9%) and Caucasians (60.3%) may limit the generalisability of findings to the true spread of the gaming community. But, similar proportions in gender have been found in prior gaming studies (e.g., Király et al., 2019). Additionally, the current study did not compare the responses of a sample who met the criteria for IGD with a subclinical or healthy gaming sample. However, the purpose of this study was an exploratory approach to understanding the dynamics of a sample with IGD specifically. Although a non-clinical sample may present lower scores in the constructs, it likely that the relationships may remain present. Finally, data collection occurred over a time period in which the COVID-19 global pandemic had potential personal consequences for individuals, which may have introduced confounding variance in the presenting psychopathology, and the motives to game, within the sample. The levels of video game use in the sample may also have been elevated in the context of requirements for some individuals to stay indoors.

### Future Directions

With consideration to the findings, implications and limitations of the current study, several recommendations have been made for the direction of future research. In particular, more stringent experiment and longitudinal-based methodologies may provide more lucrative and empirical results to support the forementioned metacognitive models. Furthermore, group comparisons between samples presenting with IGD symptomology or not may provide further evidence for the validity of the MMDTC. Alternatively, qualitative methods could highlight potential self-regulatory factors that may have not yet been considered relevant in the current models. The implementation of motivation seems to be one of these excluded but relevant factors worthy of further investigation, particularly for its impact on the cause and reasoning behind positive metacognitions as they relate to desire thinking. Implementation of preliminary metacognitive interventional (e.g., metacognitive training packages) may also prove beneficial in understanding how improving metacognitive beliefs may impact the subsequent cognitive process involved in PVGU. Finally, to the authors’ knowledge, no studies have assessed the MMDTC in a healthy sample, and future research may consider making these comparisons in all domains of the MMDTC and addiction.
